# Examples of Structural Motifs in Viral Genomes and Approaches for RNA Structure Characterization

**DOI:** 10.3390/ijms232415917

**Published:** 2022-12-14

**Authors:** Maria Nalewaj, Marta Szabat

**Affiliations:** Department of RNA Structural Genomics, Institute of Bioorganic Chemistry, Polish Academy of Sciences, Noskowskiego 12/14, 61-704 Poznan, Poland

**Keywords:** viral RNA, secondary and tertiary structures, structure prediction, high-throughput methods, cryo-EM, viruses

## Abstract

The relationship between conserved structural motifs and their biological function in the virus replication cycle is the interest of many researchers around the world. RNA structure is closely related to RNA function. Therefore, technological progress in high-throughput approaches for RNA structure analysis and the development of new ones are very important. In this mini review, we discuss a few perspectives on the structural elements of viral genomes and some methods used for RNA structure prediction and characterization. Based on the recent literature, we describe several examples of studies concerning the viral genomes, especially severe acute respiratory syndrome coronavirus 2 (SARS-CoV-2) and influenza A virus (IAV). Herein, we emphasize that a better understanding of viral genome architecture allows for the discovery of the structure-function relationship, and as a result, the discovery of new potential antiviral therapeutics.

## 1. Introduction

It is known that RNA function is closely related with its secondary and higher-order structure. RNA molecules play important roles in diverse catalytic and regulatory biological processes and can serve as disease biomarkers and therapeutic targets. Therefore, it is of great importance to understand the RNA folding properties and broaden our structure-function characterization capabilities. In RNA viruses, its genetic material is used throughout the viral replication cycle, highlighting its crucial role in the virus biology. Questions have arisen on how to best understand the RNA structure features from a biological function perspective, however, the determination of the RNA architecture is still challenging.

Many efforts have been made to study the structure, composition and organization of viral genomes. Severe acute respiratory syndrome coronavirus 2 (SARS-CoV-2) contains enveloped, non-segmented, positive-strand genomic RNA with a 5′ cap and 3′ poly-A tail that allow it to serve as an mRNA during viral protein translation. At the 5′ end, long ORF1ab poly-protein is located followed by four structural (spike, membrane, envelope, nucleocapsid) and several nonstructural proteins. In addition, coronaviruses with a positive-strand ssRNA have the largest RNA genome [1,2,3]. On the contrary, the influenza A virus (IAV) genome is a negative-strand, segmented, enveloped RNA. Eight segments encode at least eleven viral proteins and viral RNA (vRNA) is transcribed into positive-sense complementary RNA (cRNA) and messenger RNA (mRNA). The vRNA with three polymerase subunits and nucleoprotein forms viral ribonucleoprotein (vRNP) [4]. Based on the literature data, it is known that all RNA viruses characterize by a high mutation rate, which allows them to quickly adapt to the environment and evolve rapidly [3]. Therefore, it is crucial to study RNA secondary structures from IAV and SARS-CoV-2 that are conserved among viral strains [4,5].

Gaining the information on RNA architecture has a great contribution to reveal its biological function within the virus and can be useful for designing various antiviral strategies. However, vRNA dynamics and their propensity to continuous mutations are the main obstacles in designing efficient tools. Therefore, discovering the highly conserved structural elements within the genome and the development of novel methodologies are crucial to RNA structure and function characterization. Based on the available literature, we observed technological progress in the field of structural biology, especially in approaches for high-throughput structure determination. Next generation sequencing (NGS), molecular dynamics (MD) simulation and other emerging methods (including cryo-electron microscopy, cryo-EM) offer scientists the possibility to know the viral genome structural organization at increasingly high resolutions and native conditions.

This mini review summarizes a few perspectives on the viral genome structure and some approaches utilized for structural characterization. Overall, this mini review can be divided into two main parts: (i) the first concerning some viral RNA structural motifs and their potential function, and (ii) the second describing several methods to vRNA folding determination. We will discuss some recent structural studies addressing different human viruses, especially SARS-CoV-2 and IAV. Herein, we will emphasize that a better understanding of viral genome architecture allows for the discovery of structure-function relationships.

## 2. Examples of Structural Motifs within RNA Virus Genomes

### 2.1. Internal Ribosome Entry Sites (IRES) and Genome-Scale Ordered RNA Structure (GORS)

**Figure 1:**
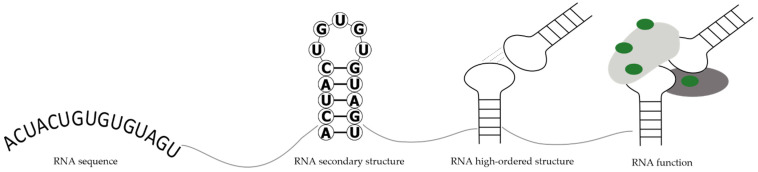
Scheme of the relationship between RNA sequence, structure and function.

The polymorphic nature and conformational flexibility of RNA facilitates their formation of a variety of structures. Many cellular processes are often regulated by RNA secondary and tertiary structures. The ability of the RNAs to fold into stable three-dimensional (3D) structures is important to RNA transcription, translation and splicing [6]. In viruses, RNA structure contributes to viral replication, evasion of host immune response, hijacking of host cell system, protein synthesis and packaging of genetic material into virions [7]. Therefore, a comprehensive understanding of RNA structures can provide important insights into its biological function in the viral life cycle. Figure 1 presents the scheme of the relationship between RNA sequence, structure, and function, which are enormously complex in the living cell.

**Figure 2:**
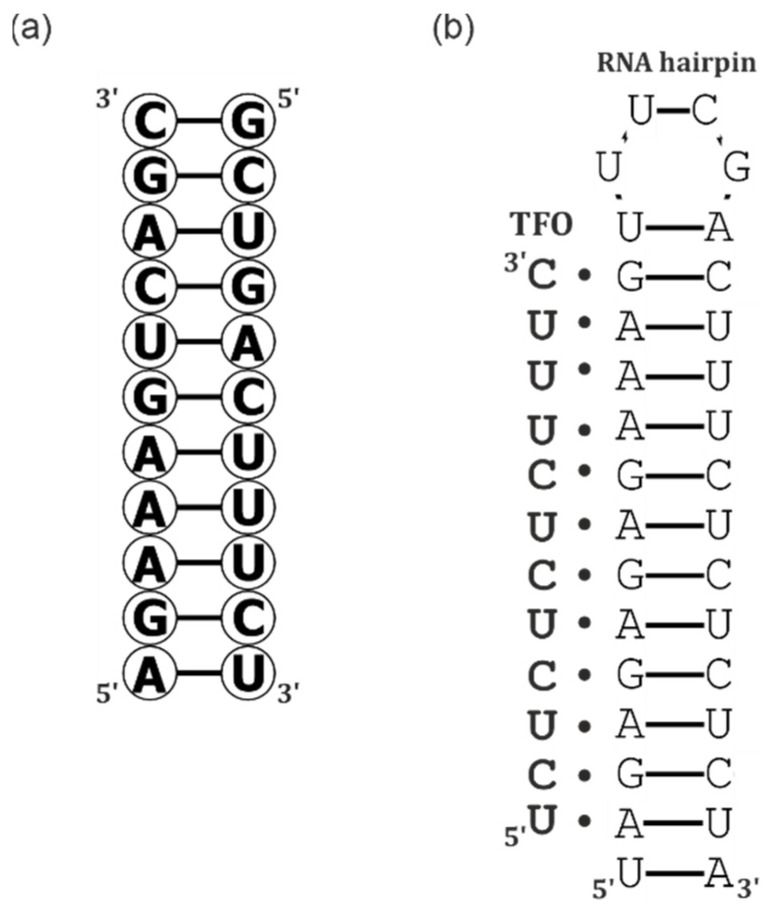
Examples of RNA secondary structures: (**a**) duplex and (**b**) triplex.

In general, RNA secondary structures consist of single-stranded and double-stranded regions, while the 3D arrangement of RNA includes helical duplexes (Figure 2a), triplexes (Figure 2b) and other components that are connected by different types of interaction. The result of these two structural levels is RNA structurome [8,9]. Interestingly, structural RNA motifs in viruses are often highly conserved among different strains and can establish complex networks of RNA-RNA interactions to shape genome architecture and control viral processes. For instance, viral RNA (vRNA) 3D structures can interact with host factors and viral proteins that have an impact on translation machinery [10]. It has been found that several short-range vRNA secondary structural motifs are likely to play important roles in virus replication [11,12,13]. Moreover, the long-range interactions formed by the partially complementary 5′ and 3′ ends of vRNA are important for the regulation of viral transcription and replication [14]. Although vRNA structure and its function are reasonably well defined and a few (mostly in vitro and bioinformatics) studies addressing the vRNA and mRNA were performed, structural studies of the vRNA, mRNA and cRNA are still in their infancy.

From the literature we know that within the viral RNA genome so-called functional RNA domains can be distinguished. Among them are the internal ribosome entry sites (IRES) playing an important role in translation initiation. They can be divided into packed IRESs that are highly conserved, extended IRESs with compact regions, flexible IRESs and inducible IRESs [15]. Various types of IRES present within the viral genome perform the same function despite different sequence and secondary RNA structure. Structural organization of IRES elements play an essential role for their activity, i.e., recruiting ribosomal subunits, initiation factors and/or RNA-protein interactions [16]. It has been described that Dicistroviridae viruses possess the IRES elements within the intergenic region of viral RNA. The architecture of the intergenic region IRESes has been well studied and found that it is highly conserved across various species of dicistroviruses [17]. Another functional RNA motifs from viral genome are cis-acting replication elements located mainly at the 3′ and 5′UTRs, but also within the coding region. These elements are involved in RNA replication and translation [18,19]. Evans and co-workers investigated these motifs in enteroviruses as conserved structural elements and determined the regions of their structure essential for activity. They also studied the influence of the genomic location of cis-acting replication elements on its function [20].

In addition to the presence of local structural motifs within the viral RNA genomes, there is an extensive RNA structure across the entire genome called genome-scale ordered RNA structure (GORS). It has been suggested that GORS is associated with viral persistence or regulates the host innate defense, potentially by shielding viral RNA from recognition in the cell [21,22]. More recently, Simmonds analyzed the coronaviruses genomes including SARS-CoV-2 for the presence of GORS and also discussed its biological purpose in the genome [22]. In other study, the authors investigated the occurrence of extensive RNA structure in the genomes of among others the Flaviviridae and Picornaviridae using large-scale thermodynamic prediction [23]. Furthermore, McFadden et al. showed that GORS can play the functional role in the genome of murine norovirus both in vitro and in vivo. They revealed that GORS potentially contributes to viral persistence in vivo [24]. As described in this paragraph, there are several specific genome secondary structures pre-sent within viral genomes that play functional role in virus biology.

### 2.2. Pseudoknots, Panhandle Motif, Kissing-Loop Motif and G-Quadruplex Structure

**Figure 3:**
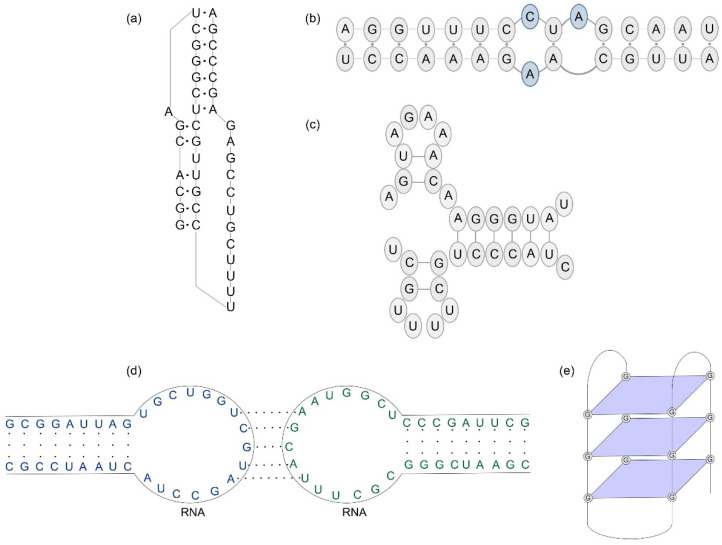
Examples of structural motifs found in the viral genomes, (**a**) pseudoknot, (**b**) panhandle, (**c**) corkscrew conformation, (**d**) kissing-loops, and (**e**) G-quadruplex.

To this day, a lot of research has been carried out to study the secondary and tertiary RNA structures of different viruses [25,26,27,28]. Viral RNA pseudoknots are known structural elements with diverse functions in virus biology (Figure 3a). As defined, a pseudoknot is formed upon base-pairing in the loop of a hairpin with a single-stranded region of complementary nucleotides elsewhere in the RNA chain. This motif is overrepresented in functional and regulatory elements of viruses and examples of pseudoknots in viral genomes are well characterized. The most common is a hairpin type (H-type) pseudoknot consisting of two helix stems (S1 and S2) and two or three single-stranded loops (L1–L3). Naturally occurring RNA pseudoknots usually forming interhelix loop contains no more than one nucleotide, and the base-paired stems tend to stack coaxially to form a quasi-continuous helix [29]. Some pseudoknots are formed upon base-pairing of single-stranded structure elements, such as bulge, interior and multibranch loops with complementary regions. It is known that the pseudoknot topology may result in many different structures that suggests the relationship between its structure and function.

For instance, Woodside and co-workers described structural dynamics of pseudoknot from SARS-CoV-2 genome showing conformers which have distinct topologies with different stability and stem-loop structures [30]. The authors found one conformer threading 5′ end and the other with unthreaded 5′ end, based on the structures observed using cryo-EM and simulations [30]. Trinity et al. investigated the structural landscape of SARS-CoV-2 ribosomal frameshifting pseudoknot using a hierarchical folding approach. They identified similarities of this motif between the novel coronavirus SARS-CoV-2, the original SARS-CoV, and the coronavirus responsible for Middle East Respiratory Syndrome [31]. Another example of pseudoknot within the viral RNA is motif found in IRES element upstream of the start codon in Senecavirus A genome [32]. This pseudoknot is predicted to be composed of two stem structures named pseudoknot stem I and II (PKS-I and -II). The authors studied the impact of motif mutations in PKS-I on viral IRES activity and on the rescue of recombinant virus. They found that mutations in PKS-I do not significantly impair the IRES activity and do not abolish luciferase expression in vitro. However, PKS-I-disrupting motifs inhibited virus rescue from mutated cDNA clones [32]. A recent study concerning the dicistrovirus 5′ IRES structural analysis has revealed the presence of pseudoknot motif relevant for proper folding and ribosome recruitment [33]. The dicistrovirus intergenic IRES adopts an overlapping triple pseudoknot RNA structure consisting of pseudoknots I, II and III (PK-I, PK-II and PK-III). This structure binds to the core of the ribosome and can regulate translation initiation [34].

Despite the flexibility and dynamics of RNA molecule, there are structural motifs that remain conserved across the strains and can serve as a potential target for antiviral therapies. One of the widely described structural motifs is a panhandle structure present in the IAV genome (Figure 3b) [4]. All eight influenza genome segments contain this motif arranged by partial base-pairing of the highly conserved 13 nucleotides at the 5′ end and 12 at the 3′ end. The panhandle region plays an important role in viral biological processes such as replication, transcription or packaging. Therefore, it became a target for antiviral compounds, e.g., small molecules. However, because it is a duplex structure, the panhandle is not easily accessible to antisense oligonucleotides or other ligands. Kesy et al. showed that panhandle motif can be bound by the dsRNA-binding PNA conjugated with neamine, what can result in reduction of viral replication [35]. Apparently, a promoter region folds into panhandle structure only in the absence of the polymerase complex, as it was recently discovered by vRNA promoter crystal structure analysis. Interestingly, it has been proposed that the viral polymerase-bound promoter region can form alternative folds. After the polymerase binding to the viral RNA termini, this region forms a secondary structure so-called ‘corkscrew’ conformation (Figure 3c). In this structural motif, the very ends of the 5′ and 3′ termini adopt intra-termini hairpin loops. The corkscrew formation has been described by various researchers to be critical element for viral polymerase activity [36,37,38,39]. Notably, the polymerase complex binding to the promoter region causes structural rearrangement and the ‘hook’ conformation in the 5′ end proximal region can be formed. The compact stem-loop (hook) structure is formed by two canonical base-pairs of the 5′ end, flanked at both sides by noncanonical A-A base pairs, leaving the 3′ end single stranded [40]. Additionally, Fodor et al. proposed the formation of the ‘fork’ model for the transcription initiation. In this structural motif base pairs are formed between nucleotide 13 at the 3′ end and 11 at 5′ end, while the viral RNA ends remain single stranded [41]. These findings show that the viral RNA promoter structure plays an important role during the viral life cycle, mainly in the transcription process.

It is worthy of note that the interacting RNA sequences can fold into stem-loops by tertiary interactions known as a ‘kissing-loop’ mechanism (Figure 3d). Many RNA-RNA complexes can be stabilized by this type of interaction. For instance, in 2017 Gamache et al. proposed a structural model of a symmetrical kissing complex [42]. In this tertiary structure occur two intermolecular interactions of complementary sequences (7 nucleotides) at the apices of two stem-loops. The authors suggest that the Ty1 RNA dimer adopts two forms, in which the kissing-loop interactions are involved. Based on these findings, this kissing complex may initiate dimerization of the retrotransposon Ty1 and packaging into virus-like particles [42]. Another example can be the study concerning the kissing-loop interaction between 5′ and 3′ ends of tick-borne Langat virus genome conducted by Pletnev and co-workers [43]. This investigation showed that destabilization of kissing-loop contacts results in the observed attenuation of virus growth, but does not affect RNA translation or its stability. The researchers suggest that the kissing-loop interaction between the genomic ends can facilitate viral RNA cyclization [43]. Moreover, since the sequences at the 5′ and 3′ loops remain invariant within the tick-borne flaviviruses, thus the kissing-loop interactions are proposed as a common mechanism in replication regulation for the group of tick-borne encephalitis virus. Furthermore, research on a functional sequence-specific interaction of the viral RNAs revealed a direct interaction between segments: polymerase-basic 1 (PB1) (nucleotides 289–309 in positive sense numbering) and non-structural (NS) (257–277) of an avian H5N2 virus [44]. Structure modeling suggested that these regions fold into hairpins, which are involved in the RNA–RNA contact through kissing-loop interactions. The application of mutagenesis confirmed that through the formation of sequence-specific interaction network, the abovementioned regions play a functional role in virion packaging. More recently, Le Sage and colleagues established a methodology termed 2CIMPL, i.e., dual crosslinking, immunoprecipitation, and proximity ligation to study the genome-wide intersegmental RNA-RNA interactions in the influenza virions [45]. The authors showed that the interactions between viral RNA segments are flexible and that individual segment can coordinate interaction with multiple other segments. Notably, they revealed that the RNA interactions of eight segments occur at multiple sites along each segment and concentrate at specific regions (hotspots) within the viral genome [45].

Among the number of structural motifs present in the viral RNA genomes, it is worth mentioning the G-quadruplex structure formation (Figure 3e). In recent years, G-quadruplexes (G4s) became the subjects of interest in many research groups [46,47,48,49,50]. These structures formed within guanine-rich (G-rich) regions consist of G-tetrads composed of planar array of four guanine residues. Many reported studies concerning the G4 structures showed that they can regulate different biological processes of various organisms. For instance, Basu and colleagues demonstrated that G4 structure can regulate the maturation of human pre-microRNA 92b [51]. The formation of above-structure lead unwinding of the canonical stem-loop structure of pre-miRNA 92b [51]. Moreover, potential G4-forming sequences (PQS) have been found in the viral RNA genomes and their potential biological functions have been studied. In the recent report, Ji et al. discovered the PQS within the RNA genome of SARS-CoV-2 located in the open reading frames of the ORF1 ab, spike, ORF3a, membrane and nucleocapsid genes [50]. Two of the PQS at positions 13385 and 24268 were confirmed to fold into RNA G4 structure by different spectroscopic methods. In addition, molecular dynamics analysis suggested the guanine bases deviating from the G-quartet planes, indicate that the G4 structure was distorted by the viral helicase nsp13. The researchers proposed targeting viral helicase and G-quadruplex structures as an attractive approach for the inhibition of SARS-CoV-2 replication [50]. Other examples are studies reported by Kowalski and co-workers, showing the presence of G4-forming sequence in the viral genome [52]. Using the combination of bioinformatics and biophysical analysis, the authors revealed the occurrence of G-rich sequences in the rhinovirus A, B and C clades, and showed that four of them are highly conserved. Furthermore, they demonstrated that pyridostatin could promote the conformational changes of G-quadruplex structure. The effect of this small molecule on the in vivo uncoating of rhinovirus has been studied. Based on the results, it has been revealed that targeting G4 structure by pyridostatin specifically inhibits the uncoating of the rhinovirus and such an approach can be a promising antiviral tool [52]. In addition to the reported papers concerning G4 structures in the viral genomes, Majee et al. reported the presence of conserved PQS motifs by analyzing the genomic Zika virus (ZIKV) sequences using a bioinformatics tool [46]. Biophysical and biochemical experiments with known G4 structure-binding ligands (N,N′-(9-(4-(dimethylamino)phenylamino)acridine-3,6-diyl)bis(3-(pyrrolidin-1-yl)propanamide) hydrochloride, BRACO-19 and 5,10,15,20-tetrakis-(N-methyl-4-pyridyl)porphine, TMPyP4) confirmed the G-quadruplex formation. Moreover, the researchers demonstrated significant inhibition of infectious ZIKV yield upon the addition of above compounds to the cell culture. Overall, they suggested that binding and stabilization of G4 structure by ligands can potentially affect virus growth, genome replication and viral protein production [46]. Similar investigations were conducted by Tomaszewska et al. presenting the G4 in the IAV genome [48]. They found the PQS motifs within segments of the IAV and evaluated their conservation among different IAV strains by bioinformatics analysis. In addition, they confirmed the propensity of these PQSs to adopt G4 folds by several biophysical methods. Taking under consideration the G-rich regions localization within the IAV RNA segments and obtained results, the authors assumed that G4 structure may play an important role in the virus life cycle [48]. As one can notice, vRNA folds into various secondary motifs, e.g., pseudoknots, kissing-loop interactions, and G-quadruplexes. Formed structures can also vary depending on viral life cycle stage, such as in the case of influenza promoter region.

## 3. Selected Approaches to Study Viral RNA Structure

### 3.1. Several Computational Tools for vRNA Structure Prediction

Viral RNA secondary and tertiary structures can be determined using the combination of computational and experimental studies. However, the flexible nature of vRNA structure makes its characterization more complex. In addition to the structural dynamics of vRNA, the length of this molecule can also be limitation, because long vRNAs can form both local or long-range interactions. Therefore, the structural analysis of such RNAs requires highly robust and reproducible methods.

To this day, many bioinformatics tools and experimental techniques have been developed for studying the RNA folding. RNA structure prediction is a mainstream approach for the structural analysis of RNA. Computational methods are based on thermodynamic calculation of minimum folding energies, phylogenetic analysis based on stochastic context-free grammars (SCFGs) and methods that combine thermodynamic and covariance detection approach. Overall, popular approaches for predicting RNA folding can be divided mainly into two categories: single-sequence and homologous-sequence methods (Table 1). The first applies the algorithms using the minimum free energy principle. Representative examples of these programs are RNAstructure [53], RNAfold [54,55], UNAfold (older Mfold) [56,57] or ScanFold [58] software. The more detailed RNAstructure includes several algorithms for RNA secondary structure prediction and analysis. This software package utilizes thermodynamics and the set of nearest neighbor parameters to predict base pairing probabilities, bimolecular structures and secondary structure common to two unaligned sequences. Moreover, RNAstructure is also useful for siRNA design or can use a number of different types of experiment mapping data (nuclear magnetic resonance, NMR, chemical/enzymatic mapping) to constrain or restrain prediction of the structure [53].

The second type of software to predict the RNA structure is based on the determination of base pairs conserved among large number of homologous sequences. An example of such a program is TurboFold for predicting secondary structures for multiple RNA sequence alignments [59]. Recently, Wang et al. proposed a novel method of prediction RNA secondary structure with pseudoknots using deep learning and improved base pair maximization principle called DMfold [60]. The DMfold tool can predict the RNA structure by deep learning similar RNA in the known structures, that uses the similar sequence instead of the homologs. Very recently, similar approach with some modifications was proposed by Xiao and co-workers to obtain a length-dependent deep learning model [61]. This RNA secondary structure prediction method with pseudoknots termed as 2dRNA (a coupled deep learning neural network for RNA) needs only the sequence information of a target RNA as an input (without the sequence alignment). The output of this method is a contact map that includes pseudoknot information [61,62]. Furthermore, Sato et. al. proposed a deep neural network-based method (MXfold2 algorithm) to predict RNA secondary structure with thermodynamic integration [63].

In the literature there are several papers describing prediction and analysis of viral RNA structural motifs using computational approaches [64,65,66]. One of such approaches is ScanFold, which has been successfully applied in structural analysis of the Zika virus [58], human herpesvirus [67] and most recently SARS-CoV-2 virus [68]. Andrews et. al. used the ScanFold tool to predict functional RNA structures and to characterize them. Expanded analyses included all sequenced RefSeq genomes allowing genome-wide comparison between human herpesviruses. All resulting data are archived and available on a public resource—the RNAStructuromeDB [67]. Very recently, interesting studies concerning RNA genome conservation and secondary structure prediction in SARS-CoV-2 and SARS-related viruses were presented by Das and co-workers [65]. The researchers determined the conservation of genome regions across SARS-CoV-2 sequences available since the COVID-19 outbreak. Moreover, they predicted additional stable structural motifs across the viral genome and provided the conserved regions in SARS-CoV-2 as potential targets for antiviral strategies. Additionally, detailed secondary structure models for the extended 5′ and 3′ UTRs, and frameshifting stimulation element were predicted from RNAz and CONTRAfold 2.0 and presented in above paper [65]. In 2018, Marz with colleagues developed a tool named SilentMutations (SIM) to analyze long-range RNA interactions within viral genomes and structured RNAs [69]. The SIM tool simulates the effect of multiple point mutations on the secondary structure, and needs only two interacting single-stranded RNAs as an input. The authors applied the SIM approach and experimentally confirmed the IAV and hepatitis C virus interactions and presented potential double mutants for in vitro characterization [69].

Advances in the field of bioinformatics and computational techniques enable the exploration of large and complex RNA structures. It is feasible to detect a long-range base pairing or intermolecular interactions, that are common in vRNAs and during vRNAs-host RNA interactions [70]. RNA 3D structure methodology offers a helpful approach to build the large 3D models of linear RNAs. For instance, the RNAcomposer system was utilized to generate 3D models of the Dengue virus RNA fragment [71]. This computer modeling used the secondary structure of RNA predicted by RNAstructure software and resultant data from the chemical mapping (selective 2′-hydroxyl acylation analyzed by primer extension, SHAPE). The researchers provided a new insight into the tertiary interactions within Dengue virus RNA structures and revealed that some motifs might function as autonomous structural element. This year, Antczak group published a paper presenting a novel webserver, named RNAspider, for topology examination and for analysis of entangled structural elements in RNA tertiary structures [72]. This tool allows automatic identification, classification and visualization of primary and higher-ordered entanglements in RNA 3D structure. The authors focused on two fragments of viral RNA genomes: UTRs from the SARS-CoV-2 genome and exoribonuclease resistant RNA from the flavivirus molecule. They used RNAspider to generate RNA 3D assembled models that present examples of entangled structure [72]. All the data presented above indicate that computational methods can be used both to predict the vRNA structure and to estimate their conservation level in the genome.

A range of RNA structure prediction tools and available resources of data have provided insight into the folding pathway of RNA molecules, including viral RNAs. Furthermore, advances in the next generation sequencing (NGS) and in the high-throughput sequencing have greatly enhanced the RNA structure determination and facilitated the large-scale structural analysis. The NGS technology is a powerful tool for massive scale RNA analysis through cDNA sequencing, also providing information on strand orientation (RNA-seq). Additionally, NGS approaches can often provide information on structure with nucleotide or near-nucleotide resolution. A typical workflow of NGS method consists of RNA fragmentation, reverse transcription into cDNA, and then library preparation according to the NGS methodology of choice. For instance, by the addition of adapters containing specific sequences interacting with the NGS platform, i.e., the surface of the flow cell—Illumina or the beads—Ion Torrent. After sequencing, bioinformatics analysis is conducted to determine the reactivity of individual nucleotides [73]. Overall, a rapid development of strategies based on the genome sequencing can facilitate investigating the virus spread and transmission, understanding the viral evolution or discovering the new antiviral drugs and molecular diagnostic tests. Recently, various HT-based technologies in combination with chemical probing have been successfully used for viral RNA structure analysis [26,45,74,75].

In order to analyze the IAV RNA structure at the single-nucleotide resolution, Dadonaite et al. used selective 2′-hydroxyl acylation analyzed by primer extension and mutational profiling method (SHAPE-MaP) [75]. This technique probes the conformational flexibility (base pairing) of each nucleotide. The researchers carried out the RNA structure analysis in both in virio and ex virio conditions. For the in virio experiments, the vRNA was probed directly inside the influenza A/WSN/1933 (H1N1) virions (in the context of vRNPs), whereas, for the ex virio, the eight RNA segments of IAV were transcribed using T7 RNA polymerase or naked viral RNA was purified from deproteinated virus particles. Based on the results, they found that viral RNA segments acquire distinct conformations and can form both intra- and intermolecular RNA-RNA interactions. Moreover, the authors used sequencing of psoralen-crosslinked, ligated and selected hybrids (SPLASH) approach for the structural analysis of interactions occurring in virio. The SPLASH method allowed them to identify the intersegment RNA interactions in the IAV genome [75]. An interesting paper describing the mapping of virus RNA-RNA interactions and RNA secondary structure within the virions was published by Zhou and Routh [26]. They developed an approach based on the NGS platform combined with the viral photoactivatable ribonucleoside crosslinking (vPAR-CL) to study the genomic viral RNA. It has been shown that vPAR-CL methodology allows for reliable identification of RNA-capsid interactions. The capsid binding sites were detected by crosslink specific uridine to cytidine transitions in NGS data. Furthermore, authors applied dimethyl-sulfate mutational profiling with sequencing (DMS-MaPseq) to study the RNA secondary structure in vivo. Based on obtained results they generated a high-resolution profile of single-stranded genomic RNA in virions and revealed that the RNA-protein binding sites favored double-stranded RNA regions [26].

In addition to the computational structure prediction tools described above, as well as HT- sequencing approaches, molecular dynamics (MD) is currently intensively expanding field. Molecular docking is a crucial technique for generating the hypotheses about structure-function relationships in RNA molecules. By computational simulation, it is possible to investigate various biomolecular processes, including conformational dynamics, ligand binding, molecules folding, and revealing the atom positions at very fine temporal resolutions. Hence, MD simulation in molecular biology or virology is an extremely helpful way to understand the molecular architecture of viral RNAs. In 2020, Selvaraj et. Al. performed structure-based virtual screening and MD simulation of SARS-CoV-2 guanine-N7 methyltransferase (nsp14) as a drug target [76]. The authors proposed a 3D model structure of SARS-CoV-2 nsp14 that contains both the guanine-N7 methyltransferase and exoribonuclease domains. Then, they focused on identification of antiviral compounds blocking guanine-N7 methyltransferase (N7-Mtase). It was found that new inhibitors can occupy and interact with the N7-Mtase binding site, which makes them potential antiviral tool against COVID-19 [76]. Among other examples of the use of MD simulations, there is a publication by Hu and colleagues [77]. Integrating MD dynamics and molecular mechanics generalized born surface area (MM-GBSA) calculation method allowed the researchers to find three natural compounds that can inhibit influenza virus RNA polymerase. The authors identified compounds which could bind tightly within polymerase acidic protein-polymerase basic protein 1 (PA-PB1) subunits and studied the nature and conformations stability of the protein-ligand complexes [77]. They suggested that the influenza virus PA-PB1 subunits interaction can be inhibited by compounds from the natural resource. The utility of molecular dynamics simulation in order to analyze vRNA structure has also been shown in the research of Anusuya et al. from 2016 [78]. In general, the Gromiha group used computational fragment-based approaches and molecular dynamics to identify dengue viral RNA-dependent RNA polymerase inhibitor. In more detail, they validated the stability of the dengue polymerase-lead complex, its binding mode, and its interaction pattern. The formation of this complex can keep the polymerase in closed conformation and results in viral replication inhibition [78]. The abovementioned examples of investigations concerning the structure-based virtual screening and MD simulations of viral RNAs underline how important is the study of the structure-function relationship for the drug discovery and development of antiviral therapies.

Overall, the improvement of existing methodology and development of effective protocols for HT techniques will enable us to investigate RNA secondary structure, as well as genome- or transcriptome-wide RNA-RNA interactions. Additionally, integrating the sequencing data obtained from HT methods with MD simulation results can provide important insights into the viral assembly process and be very helpful for understanding the infection process and pathogenesis, as well as the virus maturation in host cells.

The characterization of vRNA structural elements is a first step for rational design of antiviral agents. Nowadays, there is an urgency to identify and develop drugs against the highly pathogenic viruses. An effective and quick approach is a high-throughput screening (HTS) allowing to test existing therapeutics or identify new drug candidates. HTS identifies compounds (for example small molecules) starting from calculating the energy-minimized structures, followed by molecular docking of these structures against specific targets, for example, viral proteins. Crucial in the HTS method is performing biochemical and biological tests using a validated target representing a disease state. A complementary method is a computational screening named virtual HTS (vHTS), which computes the binding affinity of the target to the library compounds. This approach can be divided into two groups: (i) ligand-based methods that are based on the similarity between the targets and compounds, and (ii) receptor-based methods that rely on the complementarity between the compounds of interest and the target binding sites [79,80,81].

Several examples of high-throughput activity assays against different viral proteins have been described and validated [82,83,84,85,86,87]. Yang with colleagues used over 6,000 compounds in a library including approved drugs, drug candidates in clinical trials and pharmacologically active compounds, and performed high-throughput drug screening to discover new antiviral inhibitors against SARS-CoV-2 papain-like protease (PLpro) [85]. They identified four potential compounds, determined their half-maximal inhibitory concentration values and examined antiviral activities in cell-based assay. Furthermore, the crystal structure of SARS-CoV-2 papain-like protease with identified compound was presented and a unique binding mode was revealed. The solved crystal structure exposed that the interaction within the complex was stabilized through hydrophobic interactions, π-stacking interactions and hydrogen bonds [85]. In another study, a replicon-based HTS approach was subjected for the screening of ZIKV replication inhibitors [88]. This replicon system was engineered and successfully used to screen small-molecule compounds library and to identify two potential anti-ZIKV inhibitors (imidazonaphthyridine and riminophenazine). By the combination of in vitro biochemical and cellular assays, as well as in silico analysis, the researchers examined the antiviral activity of these candidates and revealed that they impair ZIKV replication, probably by interacting with the viral RdRp. Resulting data showed that replicon-based HTS approach represents useful and reliable tool for drug discovery against ZIKV infection [88]. Interestingly, Rong with collaborators discovered a novel anti-influenza virus agent (compound CBS1194) by a retroviral pseudotype-based HTS [84]. The obtained results indicated that this inhibitor can specifically target group 2 hemagglutinins and significantly inhibit virus entry during the IAV infection. Furthermore, to better understand the mechanism of action of CBS1194, biological assays and molecular docking simulations were performed. The results revealed that this compound interferes with HA-mediated membrane fusion by blocking the exposure of the fusion peptide. CBS1194 compound directly binds to a pocket near the fusion peptide and blocks the conformational change within the HA [84]. Although, the HTS strategies allow the identification and validation of agents, which target different factors involved in numerous diseases, they have some limitations and should always be supplemented by biophysical and biological studies of RNA structure. To conclude, nowadays high-throughput techniques gained popularity due to their ability to analyze the structure of a large number of sequences at the same time. By the combination of the HT approach with next generation sequencing or molecular dynamics, researchers can gain a better insight into vRNA secondary structures.

### 3.2. Several Experimental Methods for vRNA Structure Determination

As mentioned above, RNA structure prediction should be complemented by experimental validation. To improve the accuracy of computational modeling and MD simulations, several approaches for experimental RNA structure determination can be proposed [7,27,89,90]. Currently, the SHAPE method is a commonly used technique. Generally, SHAPE technology uses hydroxyl-selective electrophilic reagents reacting with ribose 2′-hydroxyl group of flexible nucleotides, which causes formation of covalent 2′-O-adduct. Then, the reverse transcriptase during reverse transcription reaction reads out the reactivity of the particular nucleotides. The synthesized cDNA is analyzed by a sequencing gel electrophoresis [7]. Over the past several years, HT methods based on chemical probing have gained popularity, because they enable studying the structure across entire viral RNA genomes by massively parallel sequencing. Technologies based on the chemical probing coupled with mutational profiling (MaP) (for example SHAPE-MaP) and next-generation sequencing (for example dimethyl sulfate mutational profiling with sequencing, DMS-MaPseq) allow for the investigation of RNA structure and its intermolecular interactions with other molecules in a native context. The SHAPE-MaP strategy requires the usage of an enzyme that introduce a change in the chemically modified site, thus internal mutations in cDNA are detected. The implementation of various alternative reagents and adduct detection methods in SHAPE probing, enables RNA folding analysis in the infected or living cells [7,91].

For instance, Heise and co-workers applied the SHAPE-MaP approach to investigate RNA secondary structure of the Chikungunya Virus (CHIKV) genome and 3′UTR variants of CHIKV [92]. Based on the SHAPE reactivity data, they analyzed base-pairing probabilities for the whole viral genome and presented RNA secondary structure model with highly specific structured regions. Additionally, the researchers also studied the flexibility and folding of CHIKV 3′UTR variants. These structural elements varied in their ability to replicate in mosquito cells. The authors revealed novel specific RNA structures in these 3′UTR variants and suggested their functional importance in viral replication [92]. In 2021, Tomezsko et al. applied DMS-MaPseq to determine the structure of HIV-1 RNA in the cells and suggest its role in splicing [93]. In this study, the authors described a new algorithm called detection of RNA folding ensembles using expectation-maximization (DREEM) and used it to directly cluster the experimental data from DMS-MaPseq (as an input for DREEM). This strategy revealed the alternative conformations that are formed by the same RNA sequences that indicates the heterogeneity of RNA structure across the entire HIV-1 genome. The role of RNA structure in HIV-1 splicing was also examined and obtained findings showed that conformational changes of RNA at critical splice sites can regulate the ration of transcript isoforms [93].

When looking for functional RNA elements within the viral genomes, different high-throughput and interactome strategies can be utilized. SHAPE mapping can be combined with crosslinking and ligation-based RNA-probing strategies. The usage of icSHAPE (in vivo click SHAPE) and PARIS (psoralen analysis of RNA interactions and structures) or SHAPE-MaP and SPLASH allowed for the discovery of secondary structure integration and function in Zika virus and dengue virus [8]. To assess the tertiary structure of SARS-CoV-2, Wan with collaborators performed comprehensive mapping of RNA interactions in vivo [94]. The characterization of RNA folding and functional structure elements along the SARS-CoV-2 genome was conducted using new methods, such as SHAPE-MaP, coupling RNA structure probing with Nanopore sequencing (abbreviated as PORE-cupine) and SPLASH. The authors obtained secondary structure models of the whole SARS-CoV-2 genome. The results showed that SARS-CoV-2 genome is highly structured inside the cells and contains many regions that are involved in intramolecular long-range interactions. Moreover, the study of subgenomic RNA (sgRNA) structure found unique conformations which are different from these in the full-length RNA genome. In addition, individual sgRNAs folding can vary despite sharing the same sequences [94].

An interesting approach, i.e., dual crosslinking, immunoprecipitation and proximity ligation (abbreviated as 2CIMPL) to identify RNA-RNA interactions of influenza virus was proposed [45]. Using this method, multiple interactions between all viral segments were presented. The analysis revealed that some intersegmental junctions are concentrated at the specific regions of IAV genome. This observation indicates that individual segments can coordinate various interactions with other segments. Furthermore, viral nucleoprotein binding to the genome does not prevent RNA-RNA interactions. All obtained results suggest that IAV genome-wide intersegmental interaction networks are complex, flexible and adjustable to sequence variations [45]. Another example of vRNA structure study is comprehensive interrogation of dengue virus RNA genome presented by Dethoff et al. [95]. The researchers first performed SHAPE-MaP analysis to generate the secondary structure of the entire DENV2 RNA genome, both in virion and ex virion. They identified some regions with well-determined structures. Then, they used the RNA interaction groups by mutational profiling (RING-MaP) approach to determine DENV2 RNA tertiary structures. The resulting data revealed numerous RNA regions with potential high-order interactions. Additionally, the analysis of structure-disrupting mutants showed the importance of these complex tertiary structure elements for global genome architecture and viral replication [95].

As it was emphasized before, numerous methodologies have been developed to characterize the architecture of viral RNA genomes. To provide complementary information to these methods, cryo-electron microscopy (cryo-EM) can be used to solve the high-resolution structures of viral assemblies. Technological progress in cryo-EM field gives scientists the opportunity of a better understanding the virus behaviors including structure organization of its genome. The EM approach includes many imaging techniques, such as cryo-electron tomography, single-particle analysis or microcrystal electron diffraction. In cryo-EM strategies, preparing samples is usually based on their immobilization in vitreous ice via rapid freezing of liquid solution that allows to keep near native cellular environment and protect from radiation damage [96,97]. The high-resolution EM analysis requires small sample amounts and can be performed in very little time. Therefore, it is commonly used in structural biology or virology.

Very recently, Zhang et al. presented the structural and functional results obtained from chemical mapping experiments, cryo-EM and biological assays (in vitro and in cellulo) [98]. They solved tertiary structure of the highly conserved frameshift stimulation element from the SARS-CoV-2 RNA genome. Based on this structure, the researchers developed antisense oligonucleotides (ASO) targeting the structural motifs of SARS-CoV-2 2 frameshift stimulation element. They demonstrated that virus replication might be impaired through modulating this element function (guided by its structure) with ASO tools. Applying cryo-EM and ASO strategy provides information how coronavirus frameshifting might be regulated and inhibited efficiently [98]. An interesting article concerning cryo-EM analysis of the Ebola virus nucleoprotein–RNA complex was written by Wolf and co-workers [99]. Researchers demonstrated near-atomic resolution structure of a recombinant NP-RNA complex expressed in mammalian cells, which was determined by the single-particle cryo-EM. Atomic model of RNA-bound NP and structures of RNA-free monomeric NP model were compared, because the transition between these two states requires conformational changes within both RNA and NP. It was suggested that coordination of nucleoprotein with RNA is sequence independent, and the transition from RNA-free state to a bound state is a dynamic process. Moreover, cryo-EM structure of the Ebola virus NP–RNA complex revealed the role of the N terminus in NP subunit oligomerization, and multiple hydrophobic and electrostatic interactions essential for viral genome encapsidation [99]. In 2021, Shan et al. used cryo-EM as the major approach to investigate the structural features of mumps virus nucleoprotein-RNA complex [100]. They found the occurrence of different assembly forms of nucleocapsid including two rings with 13 and 14 protomers, one stacked-ring filament and two nucleocapsids with distinct helical pitches. Furthermore, the resulting data indicate the conformational flexibility upon binding nucleoprotein to RNA. Structural analysis suggests that the C-terminal tail of viral nucleoprotein can regulate nucleocapsids assembly, and the C-terminal arm is speculated to be important for the transition of mumps virus nucleocapsids between the dense and hyperdense states. Overall, the application of cryo-EM approach facilitated the resolution of the high-resolution structure of different mumps virus nucleocapsids and was very helpful during increasing the knowledge of molecular mechanism for its structural plasticity [100]. To sum up, there is a constant development in experimental methods to study vRNA secondary structure. Moreover, the combination of bioinformatics tools and experimental validation is the base of NGS, MD, and HTS methods that are currently used in most of the research.

## 4. Future Perspectives

RNA function is closely related to its structure, hence, there is a continual necessity to investigate the genome structure and organization, both in vitro and in a physiological environment. Nowadays, there are numerous methods to study secondary and tertiary structures of different genomes including viruses conserved structural RNA motifs can be involved in various processes crucial for the viral replication cycle, including genome assembly and viral packaging. Therefore, accurate structural analysis of these elements should be based on an appropriate choice of modeling and experimental methods, and proper optimization and data interpretation. Although advances in RNA structure analysis provide information regarding RNA secondary and tertiary structure, much remains to be discovered and additional work is needed to evaluate the full spectrum of vRNA structure and functions in the cell.

Overall, vRNA dynamics and its propensity to continuous mutations are the main obstacles to development of efficient antiviral strategies. Therefore, finding well-described conserved structural motifs is crucial for the alternative treatment of different viral diseases in humans. vRNA can be considered a promising target for novel therapeutics. Michalak et al. suggested that structured regions of the IAV genome that are important for virus biology, can serve as a target for antisense oligonucleotides to inhibit IAV proliferation in cells [101].

Additionally, the identification of conserved structural motifs within the IAV and SARS-CoV-2 genomes can result in the development of so-called ‘programmable antiviral agents’. Various pandemic IAV strains were analyzed by SHAPE-chemical mapping and conserved motifs were discovered. Targeting critical conserved vRNA structures, by for instance LNA-modified oligonucleotides results in potent antiviral activity both in vitro and in vivo [102]. This type of ‘programmable therapy’ is extremely useful in the case of vaccine-resistant viral strains and during the period when vaccines are being developed. The knowledge of the mRNA secondary structure may also be useful in the development of mRNA vaccines, e.g., to design the vaccine against SARS-CoV-2, the structure of S-protein antigen encoded by mRNA-1273 was solved [103].

To sum up, the combination of extensive previous work and future efforts aimed at RNA structure determination and biological function identification will not only improve our basic scientific knowledge, but can also clarify the nature of the relationship between RNA structure and its functions, and further, could lead to discovering novel targets within viral genomes for efficient antiviral strategies.

## Figures and Tables

**Table 1:** Comparison of programs for RNA structure prediction.

Name	Alignment Method	Input Information	Output Information
RNAstructure	Single-sequence	Sequence and experimental data	Lowest free energy structure, base pair probabilities, siRNA design
RNAfold	Single-sequence	ssRNA sequence	RNA secondary structures
UNAfold	Single-sequence	One or two single-stranded sequences	Folding, hybridization, melting pathways simulations
ScanFold	Single-sequence	Input sequence	Functional RNA structures prediction
TurboFold	Homologous-sequence	Set of homologous RNA sequences	Secondary structures predictions for multiple RNA sequence alignments, base pairing probabilities
DMfold	Similar-sequence	Similar RNA in the known structures	RNA secondary structure with pseudoknots
CONTRAfold	Single-sequence	RNA sequence and stochastic context-free grammars (SCFGs)	Predicted RNA secondary structure
2dRNA	Single-sequence	Sequence information of a target RNA	Contact map that includes pseudoknot information
MXfold2	Single-sequence	RNA sequence and thermodynamic parameters	RNA secondary structure with thermodynamic integration
RNAz	Multiple-sequence	Processed multiple sequence alignment	Noncoding RNA detection
SilentMutations (SIM)	Multiple-sequence	Two interacting single-stranded RNAs	Effect of point mutations on the secondary structures of two interacting RNAs, long-range RNA-RNA interactions

## Abbreviations

2CIMPL	dual crosslinking, immunoprecipitation and proximity ligation
ASO	antisense oligonucleotides
Cryo-EM	cryo electron microscopy
DMS-MaPseq	dimethyl-sulfate mutational profiling with sequencing
DREEM	detection of RNA folding ensembles using expectation-maximization
G4	g-quadruplex
GORS	genome-scale ordered RNA structure
HTS	high-throughput screening
IAV	influenza A virus
IRES	internal ribosome entry sites
MaP	mutational profiling
MD	molecular dynamics
MM-GBSA	MD dynamics and molecular mechanics generalized born surface area
NGS	next generation sequencing
NMR	nuclear magnetic resonance
NS	non-structural protein
PA	polymerase acidic protein
PARIS	psoralen analysis of RNA interactions and structures
PB1	polymerase basic 1 protein
PKS	pseudoknot stem
PQS	potential quadruplex-forming sequence
RING-MaP	RNA interaction groups by mutational profiling
SARS-CoV-2	severe acute respiratory syndrome coronavirus 2
SCFGs	stochastic context-free grammars
sgRNA	subgenomic RNA
SHAPE	selective 2′-hydroxyl acylation analyzed by primer extension
SPLASH	sequencing of psoralen-crosslinked, ligated and selected hybrids
vHTS	virtual high-throughput screening
vPAR-CL	viral photoactivatable ribonucleoside crosslinking
vRNA	viral RNA
ZIKV	Zika virus
